# Interplay between Caspase 9 and X-linked Inhibitor of Apoptosis Protein (XIAP) in the oocyte elimination during fetal mouse development

**DOI:** 10.1038/s41419-019-2019-x

**Published:** 2019-10-17

**Authors:** Xueqing Liu, Veronica Castle, Teruko Taketo

**Affiliations:** 1 Department of Surgery, McGill University, RI-MUHC, Montreal, QC Canada; 2 Department of Biology, McGill University, Montreal, QC Canada; 3 Department of Obstetrics/Gynecology, McGill University, Montreal, QC Canada

**Keywords:** Apoptosis, Oogenesis

## Abstract

Mammalian female fertility is limited by the number and quality of oocytes in the ovarian reserve. The number of oocytes is finite since all germ cells cease proliferation to become oocytes in fetal life. Moreover, 70–80% of the initial oocyte population is eliminated during fetal and neonatal development, restricting the ovarian reserve. Why so many oocytes are lost during normal development remains an enigma. In Meiotic Prophase I (MPI), oocytes go through homologous chromosome synapsis and recombination, dependent on formation and subsequent repair of DNA double strand breaks (DSBs). The oocytes that have failed in DSB repair or synapsis get eliminated mainly in neonatal ovaries. However, a large oocyte population is eliminated before birth, and the cause or mechanism of this early oocyte loss is not well understood. In the current paper, we show that the oocyte loss in fetal ovaries was prevented by a deficiency of Caspase 9 (CASP9), which is the hub of the mitochondrial apoptotic pathway. Furthermore, CASP9 and its downstream effector Caspase 3 were counteracted by endogenous X-linked Inhibitor of Apoptosis (XIAP) to regulate the oocyte population; while XIAP overexpression mimicked CASP9 deficiency, XIAP deficiency accelerated oocyte loss. In the CASP9 deficiency, more oocytes were accumulated at the pachytene stage with multiple γH2AFX foci and high LINE1 expression levels, but with normal levels of synapsis and overall DSB repair. We conclude that the oocytes with LINE1 overexpression were preferentially eliminated by CASP9-dependent apoptosis in balance with XIAP during fetal ovarian development. When such oocytes were retained, however, they get eliminated by a CASP9-independent mechanism during neonatal development. Thus, the oocyte is equipped with multiple surveillance mechanisms during MPI progression to safe-guard the quality of oocytes in the ovarian reserve.

## Introduction

The number of oocytes in the ovarian reserve is finite since all germ cells cease proliferation to become oocytes in fetal life. Although the controversy over oocyte stem cells in the adult ovary remains^1–4^, it is the fact that oocytes in the ovarian reserve are exhausted with age to render reproductive senescence. In addition, 70–80% of the initial oocyte population gets eliminated during fetal and neonatal development, restricting the ovarian reserve. The cause or mechanism of this major oocyte loss remains an enigma.

The oocyte goes through Meiotic Prophase I (MPI) in fetal life, when homologous chromosomes synapse and recombine. Proper homologous recombination is essential for correct chromosome segregation at meiotic divisions, which determines the success in embryonic development. Therefore, it has been postulated that a surveillance mechanism operates to eliminate the oocytes with meiotic defects to promote the quality of oocytes in the ovarian reserve. We have previously shown that the oocyte population continuously declines throughout MPI progression, suggesting the presence of multiple causes for oocyte elimination^5^. Three major causes have been identified using mutant mice. The first cause is a failure in the repair of DNA double strand breaks (DSBs). In the oocyte, the last round of DNA replication is followed by formation of numerous DSBs, which are essential for homologous synapsis and recombination^6–8^. However, DSBs are toxic DNA lesions and must be promptly repaired using the homologous chromosome as a template. The interaction between homologous chromosomes is secured by synaptonemal complex (SC), composed of lateral and transversal elements, SCP2/3 and SCP1, respectively^9,10^. The second cause is a failure in homologous synapsis^11–15^. Transcriptional repression of unsynapsed chromatin regions, named Meiotic Silencing of Unsynapsed Chromatin (MSUC), has been proposed to determine the life or death of oocytes dependent on the repertoire of silenced genes^16,17^. These two causes may contribute to the elimination of oocytes at or beyond the pachytene stage but not at earlier stages when both DSBs and unsynapsed chromosomes are normally present in all oocytes.

The third cause for oocyte elimination is the overexpression of the Long Interspersed Nuclear Element (LINE1) gene^18^. LINE1 is usually silenced by DNA methylation, but it gets derepressed while germ cells undergo global DNA demethylation prior to entering meiosis. Overexpression of L1NE1 ORF1 protein (L1ORF1p) is associated with meiotic defects such as DNA damage, asynapsis, and unrepaired DSBs, and subsequent greater oocyte loss compared to the wild-type (WT) female. Furthermore, treatment of pregnant WT mice with a pan-inhibitor of reverse transcriptases prevents prenatal oocyte loss. This cause may explain the oocyte elimination at the pachytene stage or earlier in the WT ovary. However, it is still unclear whether physiological levels of L1ORF1p are sufficient for oocyte demise.

We anticipated that by identifying the molecular mechanism of oocyte elimination, we can block oocyte loss and then assess the oocytes which must have been eliminated in the WT ovary. We have previously reported that oocyte loss in fetal ovaries is prevented by a deficiency of Caspase 9 (CASP9), which is the hub of the mitochondrial apoptotic pathway^19^. However, CASP9 appears to be constitutively activated in most oocytes and hence not directly lead to oocyte demise. In this paper, we examined the role of endogenous cellular Inhibitor of Apoptosis Protein (IAP), which may protect oocytes from apoptotic demise in the presence of activated CASP9. Four members of the IAP family, XIAP, cIAP1, cIAP2, and NIAP, are known in mammals^20^. Of these, only XIAP inhibits cleaved CASP9, as well as effector Caspase 3 (CASP3) and Caspase 7, with a physiologically relevant potency. In the current paper, we first extended our study on the *Casp9*^−/−^ ovary. Since *Casp9*^−/−^ mice die prenatally, we could not previously follow the fate of preserved oocytes after birth. Hence, we used ovarian culture to examine *Casp9*^−/−^ ovaries at later developmental stages. Since counting oocytes in neonatal ovaries using histological sections or dissociate ovarian cells is inaccurate, we established confocal microscopic analyses of oocytes in wholemount ovaries. We then examined the role of XIAP in the oocyte population dynamics using both its null and transgenic mice in vivo.

## Methods and materials

### Animals and isolation of ovaries

All animal procedures were performed in accordance with the Canadian Council on Animal Care and approved by the McGill University Animal Care Committee. All experiments were carried out using mice, *Mus musculus*, on the C57BL/6 (B6) genetic background (Jackson Laboratory, Bar Harbor, ME, USA) including *Casp-9* null (*Casp9*^*−/−*^) (provided by Dr. T. Mak, University of Toronto), *Xiap* null (*Xiap*^*-/-*^), RING-free *Xiap* mutant (Δ*R-Xiap*) (both provided by Dr. H. Steller, Rockefeller University), and transgenic mouse in which human *XIAP* is expressed under a ubiquitin promoter (*Xiap-tg*) (provided by Dr. G. Robertson, Dalhousie University), as well as their corresponding controls. Heterozygous null males and females were crossed to generate −/−, + /−, and + / + progeny while B6 females and *Xiap-tg* males were crossed to generate heterozygous *Xiap-tg* and WT progeny. The day when a plug was observed was defined as 0.5 day postcoitum (dpc). Delivery usually occurred at 19.5 dpc, but we used dpc to define postnatal ages for consistency. Ovaries were isolated from fetal and neonatal female mice at 15.5–23.5 dpc and processed for various experiments. A piece of liver was taken from each mouse for identifying its genotype by PCR amplification, using primers as listed in Supplementary Table S1.

### Culture of fetal ovaries

Fetal ovaries isolated at 16.5 dpc were transferred individually onto Nucleopore membranes (1.0 µm pore size) floating on pre-equilibrated MEM-α medium (GIBCO 12571063) containing 10% heat-inactivated horse serum (GIBCO 26050088) and 100 U/ml penicillin/streptomycin (GIBCO 15140122) in 24-well culture dishes (Corning 353847), and incubated at 37 °C with 5% CO_2_ and humidity as previously described^21^. Ovarian explants were collected on the third day of culture or further incubated in fresh culture medium supplemented with 10 µM fulvestrant (Sigma I4409), an estrogen receptor antagonist, to simulate folliculogenesis. Two days later, half of the media was changed with fresh culture medium supplemented with fulvestrant. After two more days in culture, ovaries were collected for further analyses.

### Counting oocytes in wholemount ovaries

Ovaries isolated at 19.5–23.5 dpc were fixed in a mixture of cold methanol:DMSO (4:1) and stored at −20 °C at least overnight before immunofluorescence (IF)-staining as previously described^22^. In brief, ovaries were rehydrated in 1:1 methanol:phosphate buffered saline (PBS) for 30 min and washed thrice in holding buffer (HB: PBS containing 0.005% TritonX-100, 3% bovine serum albumin, 1% goat serum) with 1% TritonX-100 (HBT) for 1 h at room temperature. Ovaries were further incubated with the primary antibody overnight at room temperature with gentle shaking, washed thrice in HBT for 1 h, and incubated with the secondary antibody and DAPI overnight in dark at 4 °C. Ovaries were then washed thrice in PBS with 1% TritonX-100 in dark at room temperature, serially dehydrated in 25%, 50%, 75%, and 100% methanol, and finally cleared in benzyl alcohol:benzoyl benzoate (1:2) overnight at room temperature. Details of the primary and secondary antibodies are given in Supplementary Tables S2 and S3. IF-stained ovaries were imaged under a Zeiss 780 confocal microscope. Stacks were acquired at system optimized z steps between optical sections (2.49 µm intervals at ×20, ×1 or ×0.6 zoom). Quantification of oocytes was carried out using the “Surfaces” algorithm in IMARIS 8.2. After surfaces were created, overlapping objects were subtracted by the Coloc manu. Numbers of TRA98-positive cells (green), TAp63α-positive cells (red) and the cells positive for both (yellow) were counted in 3D, and the total number of oocytes was estimated by subtracting the number of both-positive cells from the sum of either green or red cells.

### Counting oocytes in microspread ovarian cells

Ovaries isolated at 18.5 or 19.5 dpc were individually transferred into 1.5 ml low-retention microfuge tubes and dissociated into single cells as previously described^23,24^. The cell suspension was incubated in a hypotonic solution (0.45 % NaCl, pH 8.0) within a QuadriPERM 4-well chamber on histology slide (Lab-Tek II 154917) for 10 min and spun down, followed by fixation and washing. The slides were vacuum dried and stored in sealed boxes with silica gel at −20 °C. The microspread ovarian cells on histology slides were incubated with the primary antibodies overnight, the secondary antibodies for 1 h, and avidin-FITC for 1 h all at room temperature. (Details of the primary and secondary antibodies are given in Supplementary Tables S2 and S3.) After IF-staining, the slides were washed, air-dried and mounted in the Prolong Gold Antifade mounting medium containing DAPI (Invitrogen P36935). The total number of germ cells was counted for each ovary using a Leica DM6000B microscope (Germany) at ×20 magnification. Around 50 cells/sample were analyzed for meiotic substages according to the IF-staining of SCP3, CREST, and γH2AFX. Localization and pattern of γH2AFX staining was also recorded. Co-localization of RAD51 was performed by omitting TRA98 while pachytene oocytes were identified by synapsis of SCP3-positive SC cores.

### Detection of L1ORF1p in microspread ovarian cells

Ovarian cell suspension was prepared from ovaries at 18.5 dpc as described above, and fixed twice in 1% formaldehyde in PBS without hypotonic treatment in a QuadriPERM 4-well chamber, followed by washing. The ovarian cells on histology slides were stained with TRA98 and anti-L1ORF1p antibodies and mounted with DAPI as described above.

### Western blotting (WB)

Ovaries at 16.5, 18.5, and 23.5 dpc were individually placed in 1.5 ml microfuge tubes, snap frozen in liquid nitrogen and kept at −80 °C until use. Two ovaries were pooled, unless specified, lysed and fractionated in SDS–PAGE according to the protocols, as previously described^19^. The proteins were then transferred electronically to a 0.23 μm nitrocellulose membrane, which was incubated with the primary antibody overnight at 4 °C and the secondary antibody conjugated with HPRT for 1 h at room temperature. The enzymatic activity was detected with a Lumi-Light ECL kit (Roche), SuperSignal™ West Femto Maximum Sensitivity Substrate (Thermo Fisher Scientific 34096) or SuperSignal West Pico PLUS Luminol/Enhancer (Thermo Fisher Scientific 34579), and visualized by autoradiography or Bio-Rad ChemiDoc XRS + system. After detection, the membranes were stripped with a ReBlot Plus Mild Antibody stripping solution (Millipore 2502) and reblotted with other antibodies. For comparing protein cleavage levels, the bands corresponding to cleaved and uncleaved forms within the same blot were quantified using the Image Lab software, and the ratio was used to indicate the relative cleavage level. To combine the results from different WB, the cleavage level in the mutant ovary was normalized against that in the WT ovary in each blot.

### Statistical analysis

All experiments were performed at least three times, and the values were presented as the mean ± SEM. In each experiment, at least three females from two litters were used, and one ovary of each pair was subjected to data analyses. Significant differences between experimental and control groups were analyzed by either Student’s *t*-test or one-way ANOVA followed by Tukey’s multiple comparison test.

## Results

### A larger number of oocytes with a higher percentage of γH2AFX staining were retained in the *Casp9*^−/−^ ovary compared to the *Casp9*^+/+^ ovary at 19.5 dpc

**Fig. 1:**
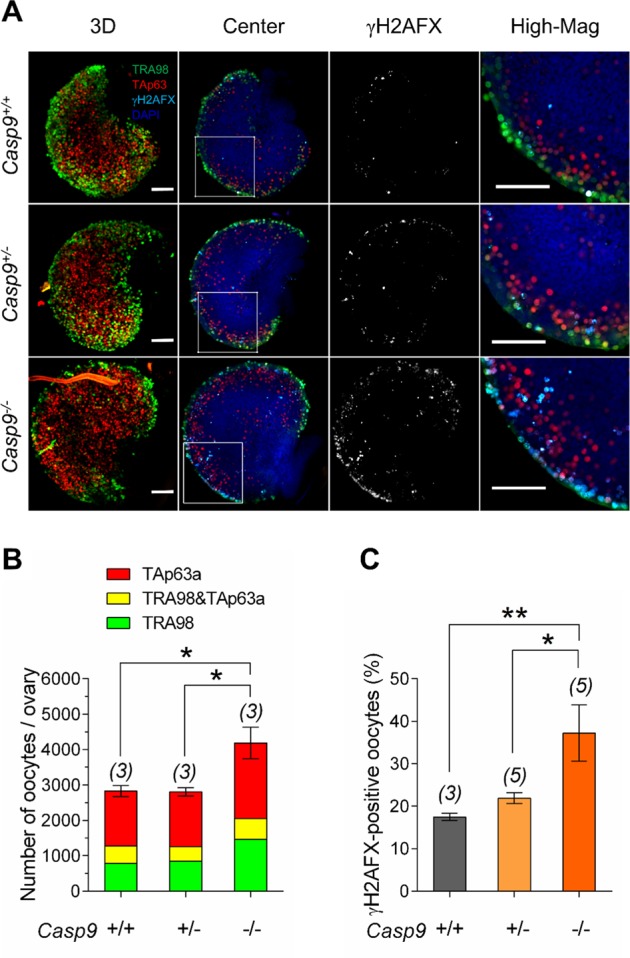
The total number of oocytes in *Casp9*^*−/−*^ ovaries compared to *Casp9*^*+/+*^ and *Casp9*^*+/−*^ ovaries at 19.5 dpc. **A** Confocal images of wholemount ovaries, IF-stained with TRA98 (green), anti-TAp63α (red), and anti-γH2AFX (cyan) antibodies with DAPI counterstaining (blue). The merged image in 3D stacks is followed by a central Z-section, γH2AFX-staining alone, and the area of the central Z-section (indicated with a white box) at a higher magnification (High-Mag). Scale bar, 100 μm. **B** The total number of oocytes per ovary. Green and red indicates the oocytes stained for TRA98 and TAp63α alone, respectively, while yellow indicates the oocytes stained for both TRA98 and TAp63α. **C** The percentage of γH2AFX-positive oocytes. Data are shown as mean ± SEM. The number of females examined is given above each column. *P < 0.05; **P < 0.01, statistical significance by *t*-test

We have previously reported a larger oocyte population retained in *Casp9*^*−/−*^ ovaries compared to *Casp9*^*+/+*^ ovaries at 19.5 dpc by counting GCNA1-positive cells in microspread ovarian cells^19^. However, this method has a disadvantage of losing many oocytes during dissociation procedure. Furthermore, GCNA1 is downregulated in oocytes by the mid diplotene stage and does not represent all oocytes at or after birth. Instead, we established a method to count the oocytes in wholemount ovaries, which were IF-stained with TRA98 and anti-TAp63α antibodies. TAp63α is expressed in the oocyte nucleus at the late pachytene stage and onwards^25,26^ whereas TRA98 recognizes the same epitope as GCNA1^27^. In *Casp9*^*+/+*^ ovaries at 19.5 dpc (Fig. 1A), TRA98-positive cells were concentrated in the peripheral region while TAp63α-positive cells were abundant in the central region, consistent with the wave of MPI progression. The average total number of oocytes (see Methods) in *Casp9*^*−/−*^ ovaries was significantly larger than that in *Casp9*^*+/−*^ or *Casp9*^*+/+*^ ovaries (Fig. 1B). Percentages of TRA98-negative, TAp63-positive cells (presumably most advanced) were comparable among the three genotypes, suggesting similar MPI progression. We also added IF-staining for γH2AFX, which indicates DNA damage and unsynapsed chromosomal regions (Fig. 1A, C). The average percentage of γH2AFX-positive oocytes in *Casp9*^*−/−*^ ovaries was significantly larger than that in *Casp9*^*+/−*^ or *Casp9*^*+/+*^ ovaries.

### Oocyte population dynamics in vivo were recapitulated in vitro, revealing CASP9-independent oocyte elimination in postnatal ovaries

**Fig. 2:**
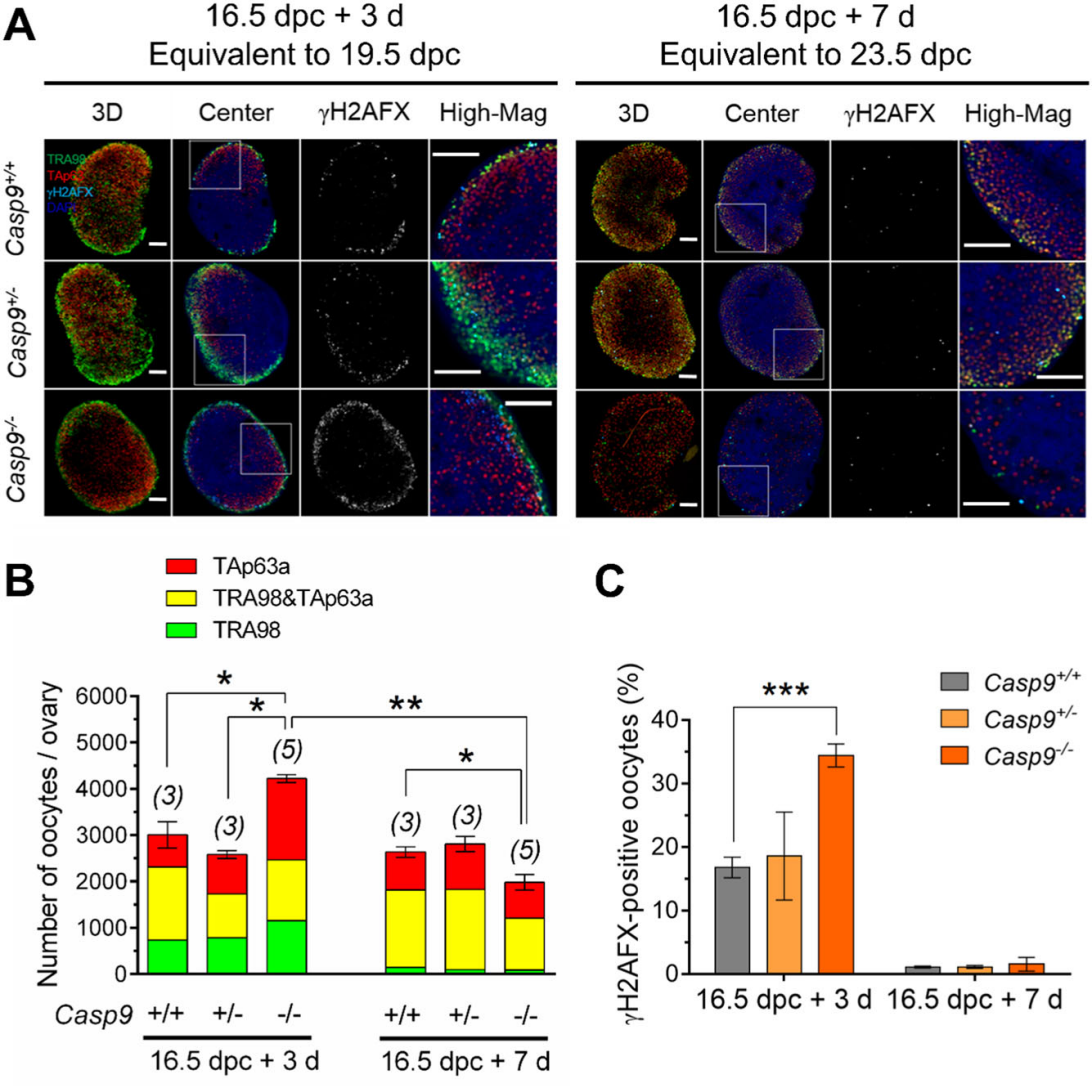
The total number of oocytes in *Casp9*^*−/−*^ ovaries compared to *Casp9*^*+/+*^ and *Casp9*^*+/−*^ ovaries isolated at 16.5 dpc and cultured for 3 or 7 days in vitro. **A** Confocal images of wholemount ovaries, IF-stained with TRA98 (green), anti-TAp63α (red), and anti-γH2AFX (cyan) antibodies with DAPI counterstaining (blue). The merged image in 3D stacks is followed by a central Z-section, γH2AFX-staining alone, and the area of the central Z-section (indicated with a white box) at a higher magnification (High-Mag). Scale bar, 100 μm. **B** The total number of oocytes per ovary. Green and red indicates the oocytes stained for TRA98 and TAp63α alone, respectively, while yellow indicates the oocytes stained for both TRA98 and TAp63α. **C** The percentage of γH2AFX-positive oocytes. Data are shown as mean ± SEM. The number of females examined is given above each column. *P < 0.05, **P < 0.01, ***P < 0.001, statistical significance by *t*-test

We next asked whether the oocytes which have circumvented apoptotic demise in *Caps9*^*−/−*^ ovaries would contribute to the ovarian reserve. Since most *Casp9*^*−/−*^ mice die before birth^28,29^, we opted for ovarian culture. To allow ovarian explants to adjust to in vitro conditions, we isolated ovaries at 16.5 dpc and cultured one of each pair for 3 days and the other for 7 days, equivalent to 19.5 and 23.5 dpc, respectively. We found that both the total number of oocytes and percentage of γH2AFX-positive oocytes in *Casp9*^*−/−*^ ovaries were larger than those in *Casp9*^*+/−*^ or *Casp9*^*+/+*^ ovaries after culture for 3 days (Fig. 2), consistent with the results in vivo (Fig. 1). No significant difference was found in the percentages of TRA98-negative, TAp63-positive cells among the ovaries of three genotypes. After culture for 7 days, the total number of oocytes in *Casp9*^*−/−*^ ovaries decreased to significantly smaller than those of other genotypes (Fig. 2). The decline in the oocyte population from the 3^rd^ to 7^th^ day in culture was significant in *Casp9*^*−/−*^ ovaries but not in other genotypes. Percentages of γH2AFX-positive oocytes were similarly low among all the genotypes. These results show that the oocyte population dynamics in vivo can be recapitulated in ovarian culture, at least, for 3 days. However, the oocytes which had been spared in *Casp9*^−*/−*^ ovaries at birth were eliminated at later developmental stages. These results suggest two phases of oocyte elimination during MPI progression although we cannot exclude the possibility that the oocytes spared in *Casp9*^*-/-*^ ovaries were more vulnerable to the culture conditions.

### The total number of oocytes was smaller in the *Xiap*^−/−^ ovary and larger in the *Xiap-tg* ovary compared to the WT ovary at 19.5 dpc

The role of XIAP in oocyte elimination was examined in *Xiap*^*−/−*^ and *Xiap-tg* ovaries. We confirmed that XIAP was undetectable in *Xiap*^*−/−*^ ovaries at 18.5 dpc by WB (Supplementary Fig. S1). The *Xiap-tg* mouse carries a human *XIAP* gene under an ubiquitin promoter^30^, and WB showed that *Xiap-tg* ovaries at both 16.5 and 19.5 dpc contained endogenous XIAP at 50 kDa and higher levels of transgenic XIAP with a Myc-tag at 75 kDa (Supplementary Fig. S2A & 2B). IF-staining showed high XIAP levels in the oocytes of both *Xiap-tg* and WT ovaries while low but distinct levels in the somatic cells of *Xiap-tg* ovaries only (Supplementary Fig. S2C). Unfortunately, our antibody against the Myc-tag did not give specific IF-staining in ovarian sections, and did not allow us to compare XIAP levels in the oocytes of the two genotypes. No difference was apparent in ovarian morphology or distribution of Foxl2-positive pregranulosa cells between *Xiap-tg* and WT ovaries at 14.5–23.5 dpc (Supplementary Fig. S3).

**Fig. 3:**
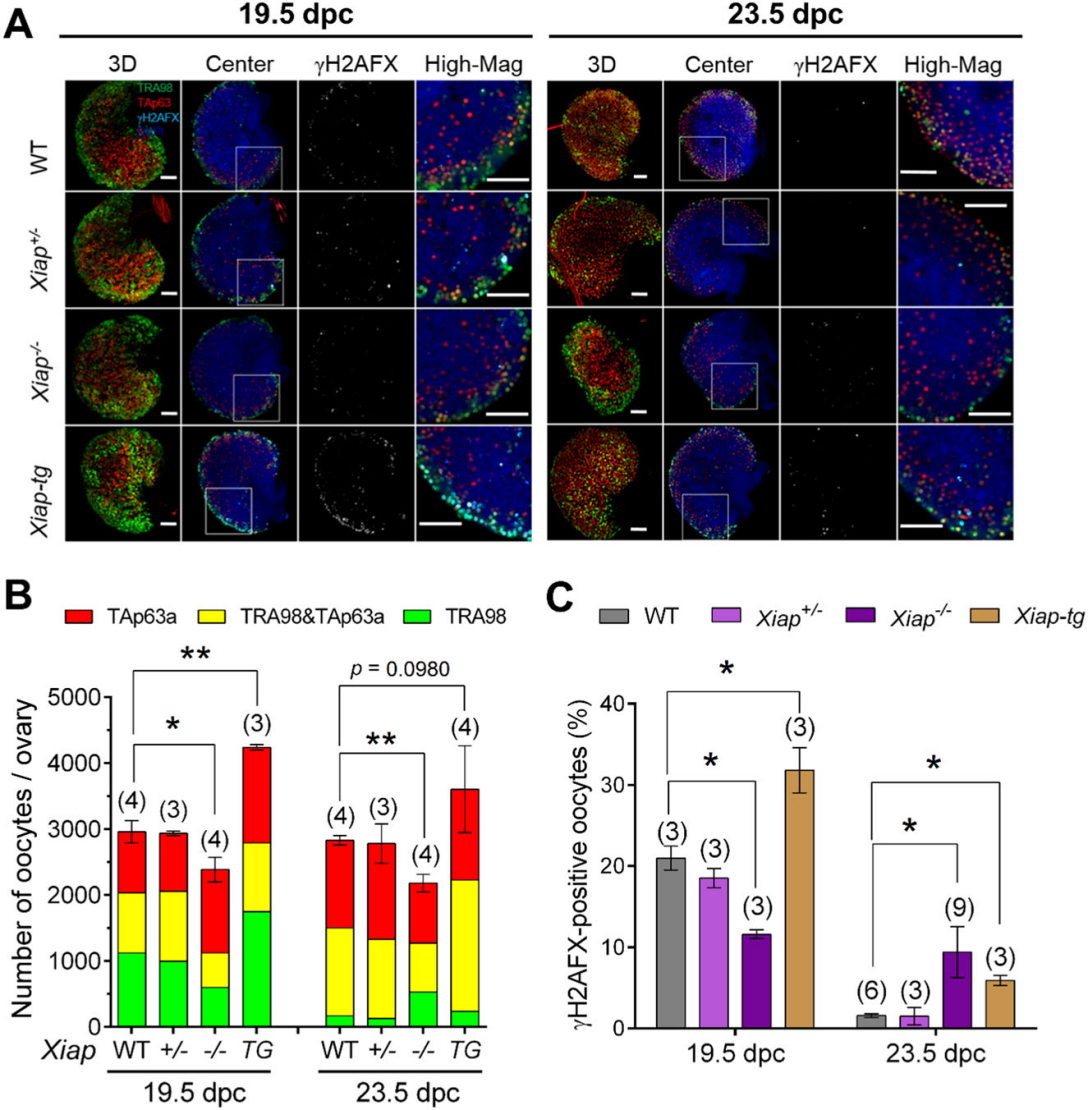
The total number of oocytes in the ovaries with XIAP deficiency or overexpression at 19.5 and 23.5 dpc. **A** Confocal images of wholemount ovaries, IF-stained with TRA98 (green), anti-TAp63α (red), and anti-γH2AFX (cyan) antibodies with DAPI counterstaining (blue). The results from *Xiap*^*+/+*^ ovaries and those from the non-transgenic ovaries were combined as WT ovaries for presentation purpose. The merged image in 3D stacks is followed by a central Z-section, γH2AFX-staining alone, and the area of the central Z-section (indicated with a white box) at a higher-magnification (High-Mag). Scale bar, 100 μm. **B** The total number of oocytes per ovary. Green and red indicates the oocytes stained for TRA98 and TAp63α alone, respectively, while yellow indicates the oocytes stained for both TRA98 and TAp63α. **C** The percentage of γH2AFX-positive oocytes. Data are shown as mean ± SEM. The number of females examined is given above each column. *P < 0.05, **P < 0.01, statistical significance by *t*-test

The results from *Xiap*^*+/+*^ ovaries and those from non-transgenic ovaries were combined as WT ovaries for presentation purpose in Fig. 3. Total numbers of oocytes counted in wholemount *Xiap*^*−/−*^ ovaries were significantly smaller than those in *Xiap*^*+/−*^ or *WT* ovaries at 19.5 and 23.5 dpc (Fig. 3A, B). On the contrary, the total number of oocytes in *Xiap-tg* ovaries was significantly larger than that in WT ovaries at 19.5 dpc, but the difference diminished to insignificant levels at 23.5 dpc. These results support our hypothesis that XIAP counteracts CASP9 to protect oocytes from apoptotic demise in fetal ovaries. Furthermore, the oocytes spared in *Xiap-tg* ovaries were eliminated after birth, although the loss was not as profound as that observed in *Casp9*^*−/−*^ ovaries in vitro. Since around 80% of the total number of oocytes in WT ovaries survived in *Xiap*^*−/−*^ ovaries at birth despite the presence of CASP9, we tested the possibility that other IAPs compensated for the XIAP deficiency by measuring the transcript levels of *cIap1* and *cIap2* in the ovaries at 18.5 dpc. However, no difference was found among WT, *Xiap*^*+/−*^, *Xiap*^*−/−*^, and *Xiap-tg* ovaries (Supplementary Fig. S4).

In accordance with the total number of oocytes, the percentage of γH2AFX-positive oocytes was smaller in *Xiap*^*−/−*^ ovaries and larger in *Xiap-tg* ovaries compared to WT ovaries at 19.5 dpc (Fig. 3A, C). In other words, the greater oocyte loss was accompanied by a loss of γH2AFX-positive oocytes in *Xiap*^*−/−*^ ovaries whereas XIAP overexpression allowed for the survival of more γH2AFX-positive oocytes in *Xiap-tg* ovaries. However, such simple correlation was not found at 23.5 dpc. The percentage of γH2AFX-positive oocytes was higher in *Xiap*^*−/−*^ ovaries despite a smaller number of oocytes compared to WT ovaries. To note, percentages of TRA98-negative, TAp63α-positive oocytes were comparable among the ovaries of all genotypes, except that the percentage in *Xiap*^*−/−*^ ovaries at 19.5 dpc was significantly larger than that in WT ovaries (*P* > 0.05) (Fig. 3B). This result may suggest that TRA98-positive oocytes were preferentially eliminated in the XIAP deficiency in fetal ovaries.

### The cleavage level of PARP1 was lower in the *Casp9*^−/−^ ovary compared to the *Casp9*^+/+^ ovary at 18.5 dpc

**Fig. 4:**
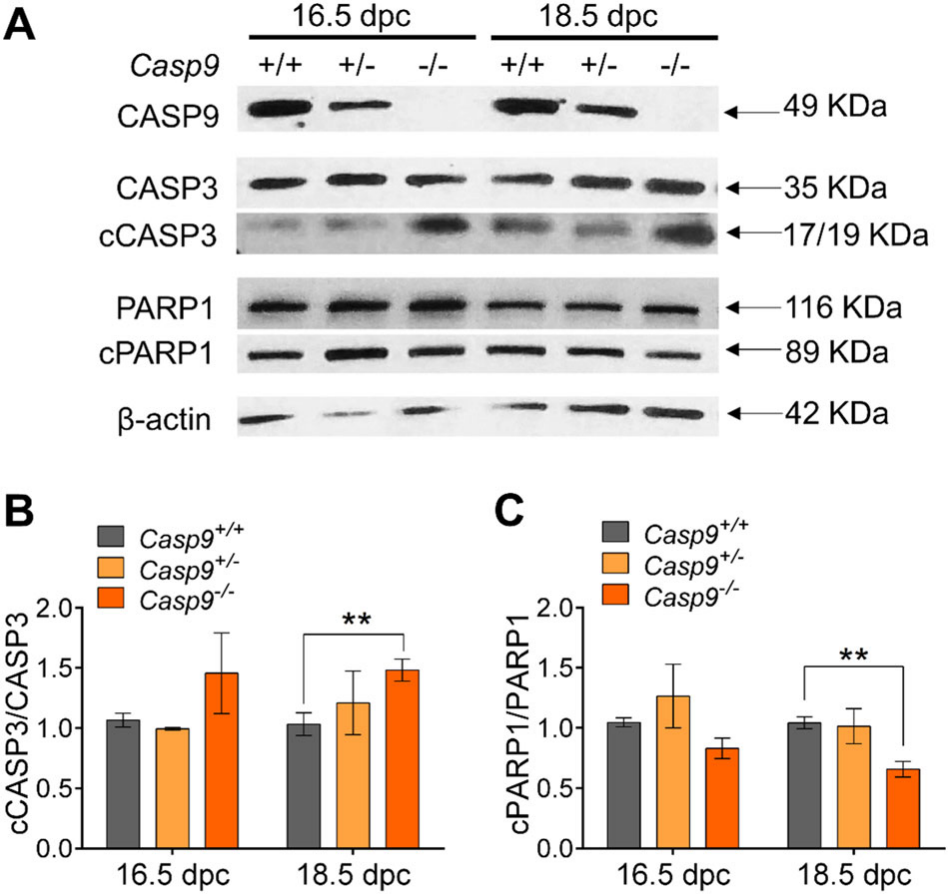
Cleavage levels of apoptosis-related proteins in *Casp9*^*+/+*^, *Casp9*^*+/−*^, and *Casp9*^*−/−*^ ovaries at 16.5 and 18.5 dpc. **A** Immunoblot of CASP9, CASP3, PARP, and β-actin as a loading control. **B** The ratio of cleaved form to full form of CASP3. **C** The ratio of cleaved form to full form of PARP1. Data are shown as mean ± SEM (*n* ≧ 3). **P < 0.01, statistical significance by *t*-test

Cleaved CASP9 activates effector CASP3 and CASP7, which in turn target various cellular components including PARP1, leading to cell demise. To define the CASP9-mediated apoptotic pathway in oocyte elimination, we examined relative cleavage levels (See Methods) of CASP3 and PARP1 in *Casp9*^*−/−*^ ovaries at 16.5 and 18.5 dpc by WB (Fig. 4). We chose 18.5 dpc to avoid the consequence of oocyte demise which was recognized at 19.5 dpc. The cleavage level of PARP1 in *Casp9*^*−/−*^ ovaries was significantly lower than that in *Casp9*^*+/−*^ or *Casp9*^*+/+*^ ovaries at 18.5 dpc as anticipated. However, the cleavage level of CASP3 in *Casp9*^*−/−*^ ovaries was significantly higher. CASP3 can be activated in many other apoptotic pathways and the CASP9 deficiency may have changed the balance between these pathways^31,32^. Alternatively, the CASP9 deficiency may have made more XIAP available for binding to and stabilizing cCASP3. However, we did not find any change in the total *Xiap* transcript or XIAP protein levels in *Casp9*^*-/-*^ ovaries compared to *Casp9*^*+/−*^ or *Casp9*^*+/+*^ ovaries (Supplementary Figure S5).

### Cleavage levels of CASP9, CASP3 and PARP1 were higher in the *Xiap*^−/−^ ovary compared to the *Xiap*^+/+^ ovary at 18.5 dpc

**Fig. 5:**
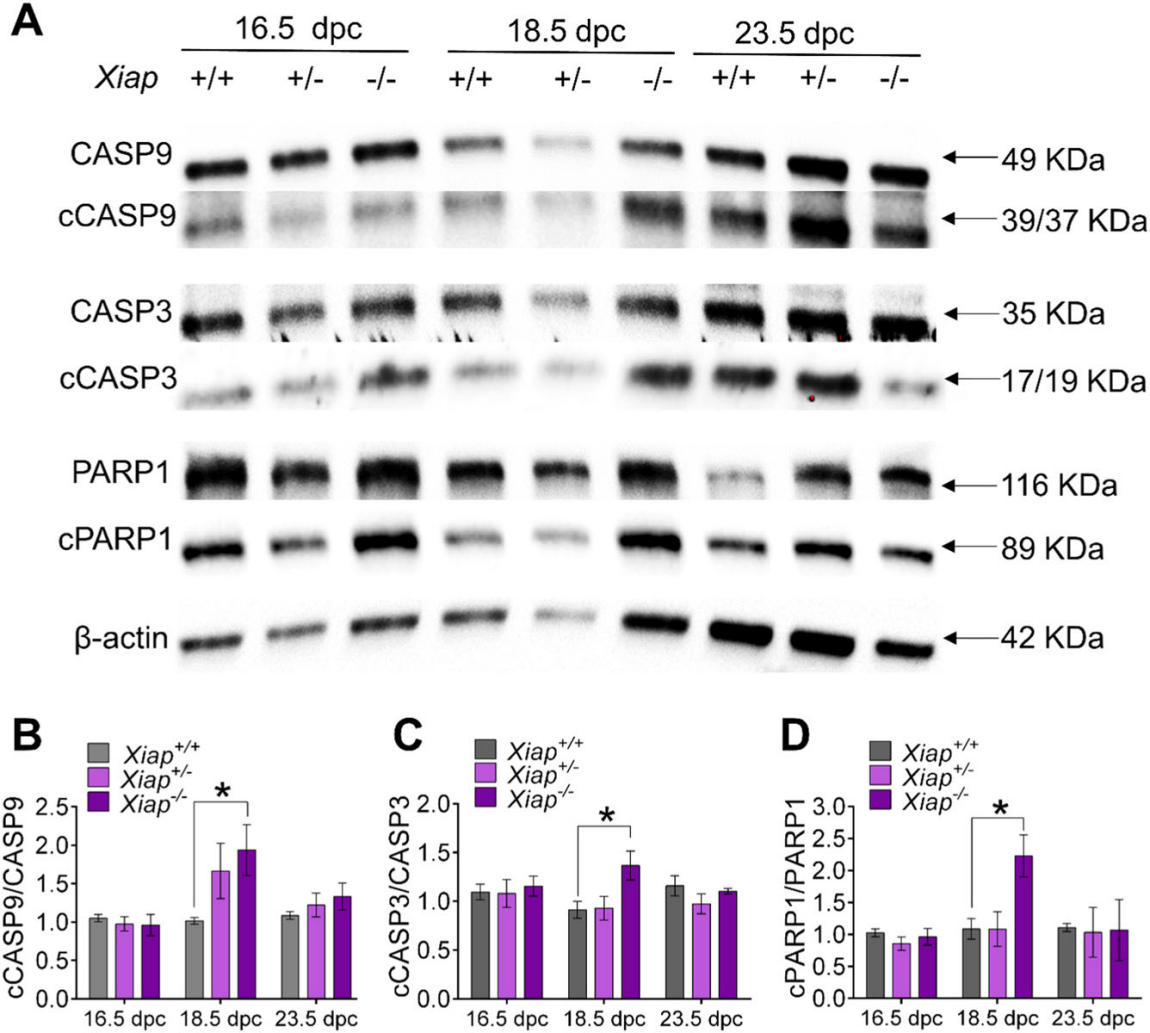
Cleavage levels of apoptosis-related proteins in *Xiap*^*+/+*^, *Xiap*^*+/−*^, and *Xiap*^*−/−*^ ovaries at 16.5, 18.5, and 23.5 dpc. **A** Immunoblot of CASP9, CASP3, PARP, and β-actin as a loading control. **B** The ratio of cleaved form to full form of CASP9. **C** The ratio of cleaved form to full form of CASP3. **D** The ratio of cleaved form to full form of PARP1. Data are shown as mean ± SEM (*n* ≧ 3). *P < 0.05, statistical significance by *t*-test

Cleavage levels of CASP9, CASP3, and PARP1 in *Xiap*^*−/−*^ ovaries were significantly higher than those in *Xiap*^*+/−*^ *or Xiap*^*+/+*^ ovaries at 18.5 dpc, but not at 16.5 or 23.5 dpc (Fig. 5). These results suggest that the XIAP deficiency transiently allowed for higher cleavage levels of CASP9, which faithfully led to higher cleavage levels of CASP3 and PARP1, as well as greater oocyte demise. This straightforward relationship between CASP9, CASP3 and PARP1 in *Xiap*^*−/−*^ ovaries contrasts the results in *Casp9*^*−/−*^ ovaries, where cCASP3 was accumulated in the presence XIAP, as described above. We considered the possibility that if XIAP inhibited cCASP9 and cCASP3 by ubiquitination, their protein levels detected by WB may not have represented cCASPs levels in action. We tested this possibility by using the *ΔR-Xiap* mouse carrying a deletion of the RING-domain required for degradation of its interacting proteins through ubiquitination^33^. If the RING-domain is required for inhibition of cCASPs in oocytes, we would expect similar effects in *Xiap*^*−/−*^ and *ΔR-Xiap* ovaries. However, cleavage levels of all CASP9, CASP3, and PARP1 in *ΔR-Xiap* ovaries were comparable with those in control ovaries (Supplementary Fig. S6), suggesting that ubiquitination is not involved in XIAP actions in oocytes.

### The oocytes retained in the *Casp9*^−/−^ ovary showed high levels of L1ORF1p expression and multiple γH2AFX foci but WT-levels of asynapsis

**Fig. 6:**
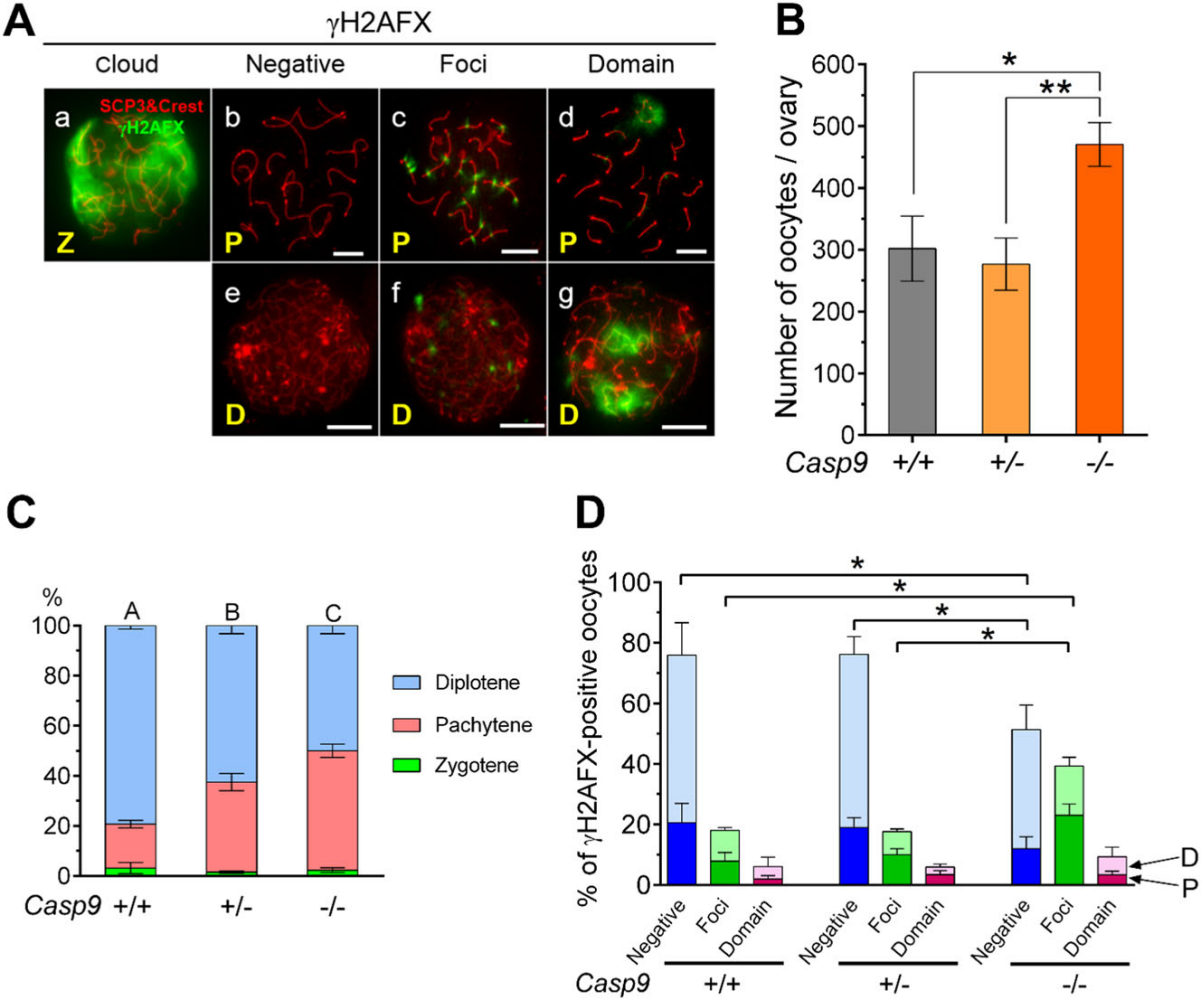
Analyses of the oocytes retained in *Casp9*^*−/−*^ ovaries at 19.5 dpc. **A** Different patterns of γH2AFX localization. MPI substages as zygotene (Z), pachytene (P), or diplotene (D) were assessed by IF-staining of SCP3 and centromeres (red). Scale bar, 10 μm. **a** Zygotene oocyte with a cloud of γH2AFX-staining over the nucleus. **b** Mid pachytene oocyte without γH2AFX signals. **c** Pachytene oocyte with multiple γH2AFX foci along SC axes. **d** Pachytene oocyte with a γH2AFX-domain over unsynapsed SCs. **e** Diplotene oocyte without γH2AFX signals. **f**. Diplotene oocyte with multiple γH2AFX foci. **g** Diplotene oocyte with two γH2AFX-domains. **B** The total number of TRA98-positive oocytes counted in microspread ovarian cells. **C** The percentage of oocytes at meiotic substages. Different capital letters indicate significant differences among the percentages of pachytene or diplotene oocytes at *P* < 0.01 by ANOVA. **D** The percentage of oocytes at the pachytene (bottom, darker color) or diplotene (top, lighter color) stage with distinct γH2AFX-staining patterns. Data are shown as mean ± SEM (*n* ≧ 3). *P < 0.05, statistical significance by *t*-test

γH2AFX accumulation indicates various normal and abnormal processes in the oocyte during MPI progression. Since the resolution is limited in wholemount ovaries, we used microspread ovarian cells with IF-staining to identify the meiotic substage, chromosome synapsis, and γH2AFX localization (Fig. 6A). With this method, the total number of TRA98-positive oocytes in *Casp9*^*−/−*^ ovaries was significantly larger than that in *Casp9*^*+/−*^ or *Casp9*^*+/+*^ ovaries at 19.5 dpc (Fig. 6B), consistent with the results using wholemount ovaries (Fig. 1B). In *Casp9*^*+/+*^ ovaries, 79.3% of oocytes reached the diplotene stage while 17.6% remained at the pachytene stage, whereas in *Casp9*^*−/−*^ ovaries, 47.7% of oocytes remained at the pachytene stage (Fig. 6C). *Casp9*^*+/-*^ ovaries showed an intermediate level, significantly different from either *Casp9*^*+/+*^ or *Casp9*^*−/−*^ ovaries. Examples of γH2AFX localization are shown in Fig. 6A. A cloud of γH2AFX signals over the entire nucleus indicated the obligatory DSBs along DNA loops, typically seen at the leptotene to zygotene stages (Fig. 6Aa). Such γH2AFX signals gradually disappeared by DSBs repair along chromosome synapsis by the mid pachytene stage (Fig. 6Ab and e). However, residual γH2AFX signals were often seen at the early pachytene stage or punctate γH2AFX foci remained along SC axes even at the mid pachytene or early diplotene stage (Fig. 6Ac and f). An intense γH2AFX domain was usually associated with unsynapsed SC regions (Fig. 6Ad and g). As summarized in Fig. 6D, the percentage of oocytes with γH2AFX multiple foci at the pachytene and diplotene stages, either combined or separately, was significantly larger in *Casp9*^*−/−*^ ovaries than in *Casp9*^*+/−*^ or *Casp9*^*+/+*^ ovaries. By contrast, percentages of oocytes with γH2AFX domains were comparable among the ovaries of all genotypes, suggesting that the oocytes with synaptic errors were not targeted for the CASP9-dependent apoptotic elimination. We conclude that the oocytes with locally unrepaired DSBs at the pachytene stage were accumulated in *Casp9*^*−/−*^ ovaries at 19.5 dpc.

We next asked whether multiple γH2AFX foci persisted in *Casp9*^*−/−*^ oocytes due to overall inefficient DSB repair. It is known that 3′-hang of DSBs are recognized by RAD51 and DMC1, which facilitate DNA strand invasion and repair by using the homologous DNA strand^34^. Accordingly, ovaries from *Casp9*^*−/−*^ and *Casp9*^+/+^ females at 18.5 and 19.5 dpc were subject to microspread cell preparation and IF-staining for TRA98, RAD51 and SCP3. Numerous RAD51 foci were seen along SC axes at the zygotene stage and much fewer at the pachytene stage except that RAD51 foci remained at a higher density over both γH2AFX domains and multiple foci along SC axes in both *Casp9*^*−/−*^ and *Casp9*^+/+^ ovaries (Supplementary Fig. S7). Thus, the overall DSBs repair was efficient but γH2AFX foci with locally unrepaired DSBs persisted in *Casp9*^−*/−*^ oocytes.

**Fig. 7:**
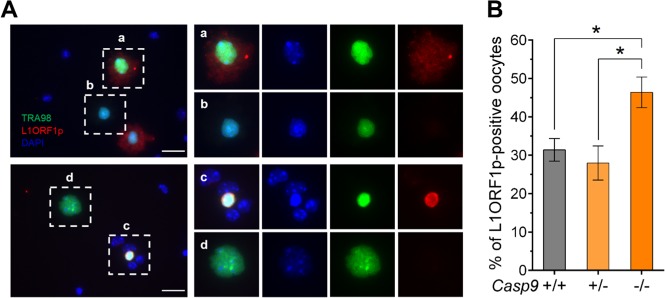
IF-staining of L1ORF1p in the oocytes retained in *Casp9*^*−/−*^ ovaries at 18.5 dpc. **A** IF-staining for TRA98 (green) and L1ORF1p (red) with DAPI nuclear staining (blue) in the microspread ovarian cells without hypotonic treatment. Scale bar, 25 μm. The area indicated with a white box is shown at ×4 magnification on the right panel, in the order of merged, DAPI alone, TRA98 alone, and L1ORF1p alone. **a** L1ORF1p staining in the oocyte cytoplasm. **b** No L1ORF1p staining in an oocyte harboring a pyknotic nucleus. **c** Intense L1ORF1p staining in a pyknotic nucleus. **d** No L1ORF1p staining in the entire oocyte. **B** The percentage of oocytes with L1ORF1p IF-staining in either the nucleus or cytoplasm. Data are shown as mean ± SEM (*n* ≧ 3). *P < 0.05, statistical significance by *t*-test

We also tested whether LINE1 expression was associated with the oocyte demise by the CASP9-depenent apoptotic pathway^18^. The dissociated cells without hypotonic treatment from the ovaries at 18.5 dpc were subject to IF-staining for TRA98 and L1ORF1p (Fig. 7A). L1ORF1p staining was seen in the cytoplasm of some oocytes (Fig. 7Aa) and occasionally in pyknotic nuclei (Fig. 7Ac), although not all pyknotic nuclei were positive (Fig. 7Ab). The percentage of L1ORF1p-positive oocytes in *Casp9*^*−/−*^ ovaries was significantly larger than that in *Casp9*^*+/−*^ or *Casp9*^*+/+*^ ovaries (Fig. 7B). We conclude that the oocytes with LINE1 overexpression were targeted for elimination by the CASP9-dependent apoptotic pathway.

## Discussion

Apoptosis has been implicated as the mechanism of oocyte elimination during fetal and neonatal ovarian development in the mouse^35–38^. However, in most previous studies, the total number of oocytes was counted only in postnatal ovaries, obscuring when and how the oocyte population was eliminated. Our current study aimed at clarifying the role of interplay between CASP9 and XIAP in oocyte elimination during different phases of ovarian development.

### Oocyte surveillance during fetal ovarian development

Our results show that over 30% of oocytes were spared from apoptotic elimination at birth in the CASP9 deficiency. Thus-retained oocytes appeared surprisingly normal; DSBs were largely repaired and the frequency of asynapsis was under 7%, comparable to that in WT ovaries. The only abnormality recognized is the accumulation of oocytes at the pachytene stage with L1ORF1p enrichment and persistent multiple γH2AFX foci. This is not due to a delay in MPI progression since the percentage of zygotene oocytes was comparable to WT ovaries. We conclude that high levels of LINE1 expression are associated with oocyte demise by the CASP9-dependent apoptotic pathway. Persistent multiple γH2AFX foci may be a consequence of LINE1 overexpression but unlikely the cause for oocyte elimination at this phase. Severer defects in the oocytes with LINE1 overexpression in a MAEL deficiency were previously reported^18^. Interestingly, these authors found that 45% of oocytes were spared from 15.5 to 18.5 dpc when LINE1 expression was suppressed, in close agreement with our current results.

### Interplay between CASP9 and XIAP in the regulation of oocyte population

Our current results indicate that XIAP counterbalances cCASP9 to prevent the cleavage of CASP3 and PARP1 and the consequent oocyte demise. This XIAP action does not involve the Ring-domain or ubiquitination, and hence is likely mediated by its direct binding to cCASP9 and cCASP3^39,40^. Therefore, the increased cleavage level of CASP9 in *Xiap*^*−/−*^ ovaries that we observed (Fig. 5) cannot be explained by a prevention of ubiquitination. As many oocytes survived in *Xiap*^*−/−*^ ovaries despite the presence of cCASP9, we considered compensatory roles of other cIAPs^41,42^, but did not find a difference in their transcript levels. We have not yet excluded changes at their protein levels. Nonetheless, we speculate that the oocytes which are destined to survive may be protected from apoptotic demise by multiple mechanisms.

In *Casp9*^*−/−*^ ovaries, the cleavage level of PARP1 and oocyte demise were decreased as predicted. However, the cleavage level of CASP3 was rather increased. By contrast, in *Xiap*^*−/−*^ ovaries, the higher cleavage level of CASP9 was consistent with cleavage levels of CASP3 and PARP1, as well as oocyte demise. We speculate that the CASP9 deficiency made more XIAP available for stabilizing CASP3. Alternatively, the CASP9 deficiency may have upregulated other pathways involving CASP3^31,32^. In the current study, all WB were performed using ovarian lysate. Furthermore, all mutants were conventional knock-out mice. Therefore, we cannot exclude the contribution of somatic cells in WB results. However, both cCASP9 and XIAP are detectable mainly within the oocytes^19^ (current study) while somatic cells do neither actively proliferate nor die in fetal ovaries, unlikely impacting the overall results. Importantly, the results using various mutants were consistent to support the role of CASP9-XIAP interplay in oocyte elimination. Therefore, we believe that a change in the oocyte population was largely reflected into WB results. Nonetheless, further studies are needed to clarify the molecular interaction between XIAP, CASP9, and CASP3 in oocytes.

### Oocyte surveillance during postnatal ovarian development

The oocytes spared in *Casp9*^*−/−*^ ovaries at birth were swiftly eliminated in postnatal development in culture. This finding is consistent with the report that the oocytes accumulated by inhibition of LINE1 expression were lost after birth^18^. The oocytes spared in *Xiap-tg* ovaries were also diminished by 23.5 dpc, but to a lesser extent than in *Casp9*^*−/−*^ ovaries. This difference can be attributed to the oocyte population dynamics in vivo vs. in vitro. It is also possible that XIAP overexpression suppressed CASP3 that contributed to oocyte loss beside the CASP9-dependent pathway. Regardless of the mechanism, the oocytes that had circumvented the CASP9-dependent apoptotic demise were eliminated by a CASP9-indepdendent mechanism that became available in postnatal ovaries. It is well established that the oocytes with unrepaired DSBs or unsynapsed chromosomes are eliminated by the CHK2-TAp63 apoptotic pathway, which becomes active only after birth^25,26,43–45^. It remains to be determined if the CHK2 pathway can operate in the CASP9 deficiency. Autophagy is an alternative mechanism for oocyte demise^46,47^. In fact, apoptosis and autophagy share many molecules such as XIAP and CASP3^48^. However, we found no difference in the LC3I/LC3II ratio, a hallmark of autophagy^49^, between *Casp9*^*−/−*^ and *Casp9*^*+/+*^ ovaries after culture (data not shown). Necroptosis is known to operate under the apoptosis deficient conditions^50,51^. Examples are so far limited to the apoptosis initiated by extrinsic causes. Further studies are needed to identify the mechanism of CASP9-indepdendent oocyte elimination in postnatal ovaries.

### Summary

Our current results revealed two phases of oocyte surveillance during MPI progression. The first phase takes place during fetal development, during which oocytes undergo DSB formation and homologous synapsis, dependent on the CASP9-mediated apoptotic pathway. The oocytes that have survived in the CASP9 deficiency appear to carry high L1ORF1p expression levels and locally unrepaired DSBs along SC axes, but no defect in overall DSB repair or synapsis. XIAP appears to counterbalance the CASP9 activity to regulate the oocyte population. The second phase of oocyte surveillance takes place during neonatal development, during which oocytes pass the pachytene stage and reach the diplotene resting stage, independent of CASP9. These results suggest that the oocyte is equipped with multiple surveillance mechanisms during MPI progression to safe-guard the quality of oocytes in the ovarian reserve.

## Supplementary information

Supplemental Material
