# Supplementary material for: Interplay between Caspase 9 and X-linked Inhibitor of Apoptosis Protein (XIAP) in the oocyte elimination during fetal mouse development
Source: Cell Death Dis. 2019 Oct 17;10(11):790. doi: 10.1038/s41419-019-2019-x (PMC6797809; doi:10.1038/s41419-019-2019-x)
Supplement: Supplementary file 1 — Supplemental Material [file 41419_2019_2019_MOESM1_ESM.docx]

**Supplemental Material**

**Interplay between Caspase 9 and X-linked cellular Inhibitor of Apoptosis Protein (XIAP) in the oocyte elimination during fetal mouse development**

Xueqing Liu^1^, Veronica Castle^2^, Teruko Taketo^1, 2, 3*^

^1^Department of Surgery, ^2^Department of Biology, and ^3^Department of Obstetrics/Gynecology, McGill University, Montreal, Quebec

* Correspondence: Teruko Taketo, Department of Surgery, Research Institute of McGill University Health Centre, 1001 Decarie Blvd, Montreal, Quebec, Canada H4A 3J1, e-mail: [teruko.taketo@mcgill.ca](mailto:teruko.taketo@mcgill.ca)

Table S1. Primers used for DNA-PCR genotyping

Table S2. Primary antibodies used for immunofluorescence staining and western blotting

Table S3. Secondary antibodies used for immunofluorescence staining and western blotting

Table S4. Primers used for qRT-PCR

Fig. S1. XIAP protein levels in *Xiap^-/-^*, *Xiap^+/+^*, and *Xiap^+/-^* ovaries at 18.5 dpc.

Fig. S2. *Xiap* expression levels in WT and *Xiap-tg* ovaries at 16.5 and 19.5 dpc.

Fig. S3. Ovarian morphology in WT and *Xiap-tg* females at 14.5-23.5 dpc.

Fig. S4. Transcript levels of *cIap1* and *cIap2* in WT, *Xiap^-/-^* and *Xiap-tg* ovaries at 18.5 dpc.

Fig. S5. Expression levels of IAP family genes in *Casp9^+/+^*, *Casp9^+/-^* and *Casp9^-/-^* ovaries at 16.5 and 18.5 dpc.

Fig. S6. Cleavage levels of CASP9, CASP3 and PARP1 in *ΔR-Xiap^+/+^*, *ΔR-Xiap^+/-^* and *ΔR-Xiap^-/-^* ovaries at 16.5, 18.5 and 23.5 dpc.

Fig. S7. Localization of RAD51 in the oocytes retained in *Casp9^-/-^* ovaries at 18.5 dpc.

Table S1. Primers used for DNA-PCR genotyping

| **Gene** | **Primers (5’-3’)** | **Amplicon Length** | **Genotype** |
| --- | --- | --- | --- |
| *Casp9* | F: CTTTGTCCCTCCTGTTGTGTCTTCA  R: CAGAGCGAGAATGAAGGGGAAACAA | 400 bp | WT |
| *Casp9* | F: CTTATGTATTCCCGAGCCCGTGGTA  R: GTATGCTATACGAAGTTATTAGTCC | 600 bp | *Caps9*^-^ |
| *Xiap* | F: TGGAGAGTTTGTTGAATTTGGG  R: TGGGAAATAGAAATCCTTTTGC | 400 bp | WT |
| *Xiap* | F: TTTGAAGTTCCTAATGCAATGTTCTC  R: ATCGAGCGAGCACGTACTTCGGATG | 850 bp | *Xiap*^-^ |
| *ΔRXiap* | F: TAAAGCCTTTACCTTCTTCTCTATTTCC  R: TGGGACAGGTAGGATTTAGTGCTTCG | 100 bp  200 bp | WT  *ΔR-Xiap* (FRT) |
| *UbXiap* | F: GGATCCTCTGATGCTGTGAGTTCTGATAGGAATTTCCC  R: GACTCGAGCTAAGTAGTTCTTACCAGACACTCCTCAAG | 350 bp  absent | *UbXiap*  WT |

Table S2. Primary antibodies used for immunofluorescence staining and western blotting

| **Primary Antibody** | **Host Species** | **Source/Company** | **Catalog Number** | **Concentration** |
| --- | --- | --- | --- | --- |
| DDX4/MVH | Rabbit | Abcam | ab13480 | 1:500 |
| TRA98 | Rat | B-Bridge | 73-003 | 1:1000 |
| XIAP | Rabbit | Cell Signaling | 2042 | 1:1000 |
| Caspase-9 | Rabbit | Cell Signaling | 9504 | 1:1000 |
| Caspase-3 | Rabbit | Cell Signaling | 9662 | 1:1000 |
| Cleaved Caspase-3 | Rabbit | Cell Signaling | 9661 | 1:1000 |
| PARP1 | Rabbit | Cell Signaling | 9542 | 1:1000 |
| Cleaved PARP1 | Mouse | Cell Signaling | 9548 | 1:1000 |
| Cleaved PARP1 | Rabbit | Cell Signaling | 9544 | 1:500 |
| TAp63α | Rabbit | Cell Signaling | 13109 | 1:50 |
| γH2AFX | Chicken | Biorbyt | Orb195374 | 1:2000 |
| γH2AFX | Mouse | Millipore | 05-636 | 1:2000 |
| RAD51 | Rabbit | Abcam | Ab133534 | 1:500 |
| L1ORF1p | Rabbit | Alex Botvin | gift | 1:500 |
| CREST | Human | ImmunoVision | HCT-0100 | 1:2000 |
| SYCP3 | Rabbit | Abcam | Ab15093 | 1:1000 |
| SYCP3 | Mouse | Abcam | Ab97672 | 1:1000 |
| FOXL2 | Goat | Novus | NB100-1277 | 1:500 |
| Myc-tag | Mouse | Millipore | 05-419 | 1:1000 |

Table S3. Secondary antibodies used for immunofluorescence staining and western blotting

| **Secondary Antibody** | **Host Species** | **Source/Company** | **Catalog Number** | **Concentration** |
| --- | --- | --- | --- | --- |
| Anti-mouse FITC | Goat | Jackson | 155-095-003 | 1:1000 |
| Anti-mouse Rhodamine | Goat | Invitrogen | R6393 | 1:1000 |
| Anti-rat Alexa Fluor 488 | Goat | Invitrogen | A-11006 | 1:1000 |
| Anti-chicken Alexa Fluor 647 | Goat | Biorbyt | Orb195374 | 1:2000 |
| Anti-rabbit FITC | Goat | Jackson | 111-095-003 | 1:1000 |
| Anti-rabbit Rhodamine | Goat | Invitrogen | 31670 | 1:1000 |
| Anti-mouse biotin | Goat | Invitrogen | 31800 | 1:1000 |
| Anti-rabbit biotin | Goat | Invitrogen | 31823 | 1:1000 |
| Streptavidin-rhodamine |  | Invitrogen | S6366 | 1:1000 |
| Anti-rabbit-HRP | Goat | Cell Signaling | 7074S | 1:2000 |
| Anti-rat-HRP | Goat | Cell Signaling | 7077S | 1:2000 |
| Anti-mouse-HRP | Goat | Invitrogen | A16072 | 1:10000 |

Table S4. Primers used for qRT-PCR

| **Gene** | **Primers (5’-3’)** | **Amplicon Length** |
| --- | --- | --- |
| *GAPDH* | F: CCTGGAGAAACCTGCCAAGTAT  R: TGAAGTCGCAGGAGACAACCT | 117 bp |
| *Xiap* | F: CCATGTGTAGTGAAGAAGCCAGAT  R: GATCATCAGCCCCTGTGTAGTAG | 114 bp |
| *cIap-1* | F: TGGATCGCAATGATGATGTC  R: GAAACCATTTGGCGTGTTCT | 99 bp |
| *cIap-2* | F: CGAGGAGGAGGAGTCAGATG  R: GGAGGCAATACAGCATTGGT | 98 bp |

No XIAP protein is detectable in the *Xiap^-/-^* ovary at 18.5 dpc.


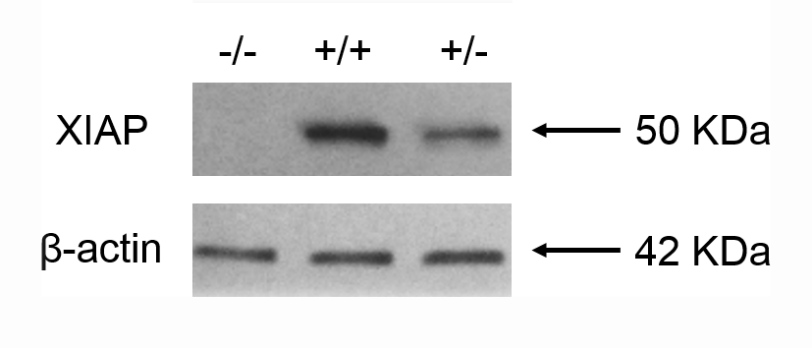


Fig. S1. WB of XIAP proteins in *Xiap^-/-^*, *Xiap^+/+^*, and *Xiap^+/-^* ovaries at 18.5 dpc.

The *Xiap-tg* mouse expresses the human *XIAP* gene under an ubiquitin promoter. Human *XIAP* protein levels were moderately higher than endogenous mouse XIAP levels in the *Xiap-tg* ovary at 19.5 dpc. XIAP was localized in the oocytes in the WT ovary whereas XIAP is detectable in both oocytes and somatic cells in the *Xiap-tg* ovary.

**B**


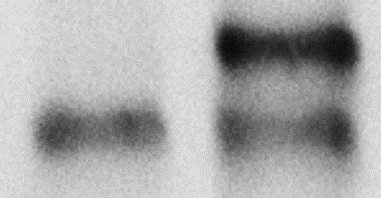

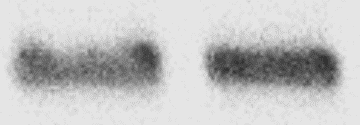


Anti-β-actin

Anti-XIAP

WT *Xiap-tg*

19.5 dpc

75 KDa

50 KDa

42 KDa

16.5 dpc

**A**

WT *Xiap-tg*


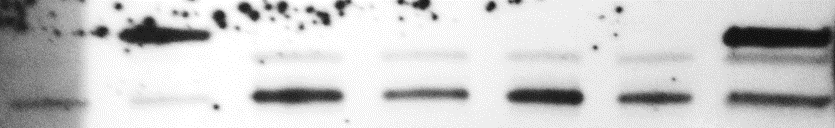


75 KDa

Anti-cMyc


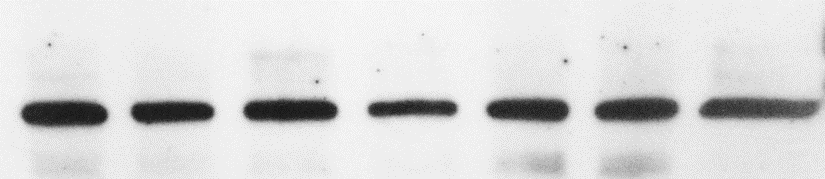


Anti-XIAP

50 KDa


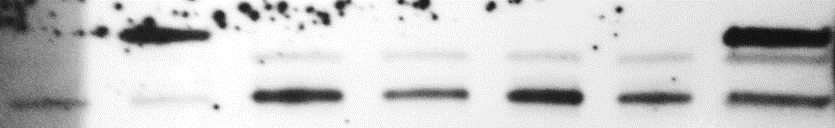


Anti-β-actin

42 KDa

**C**


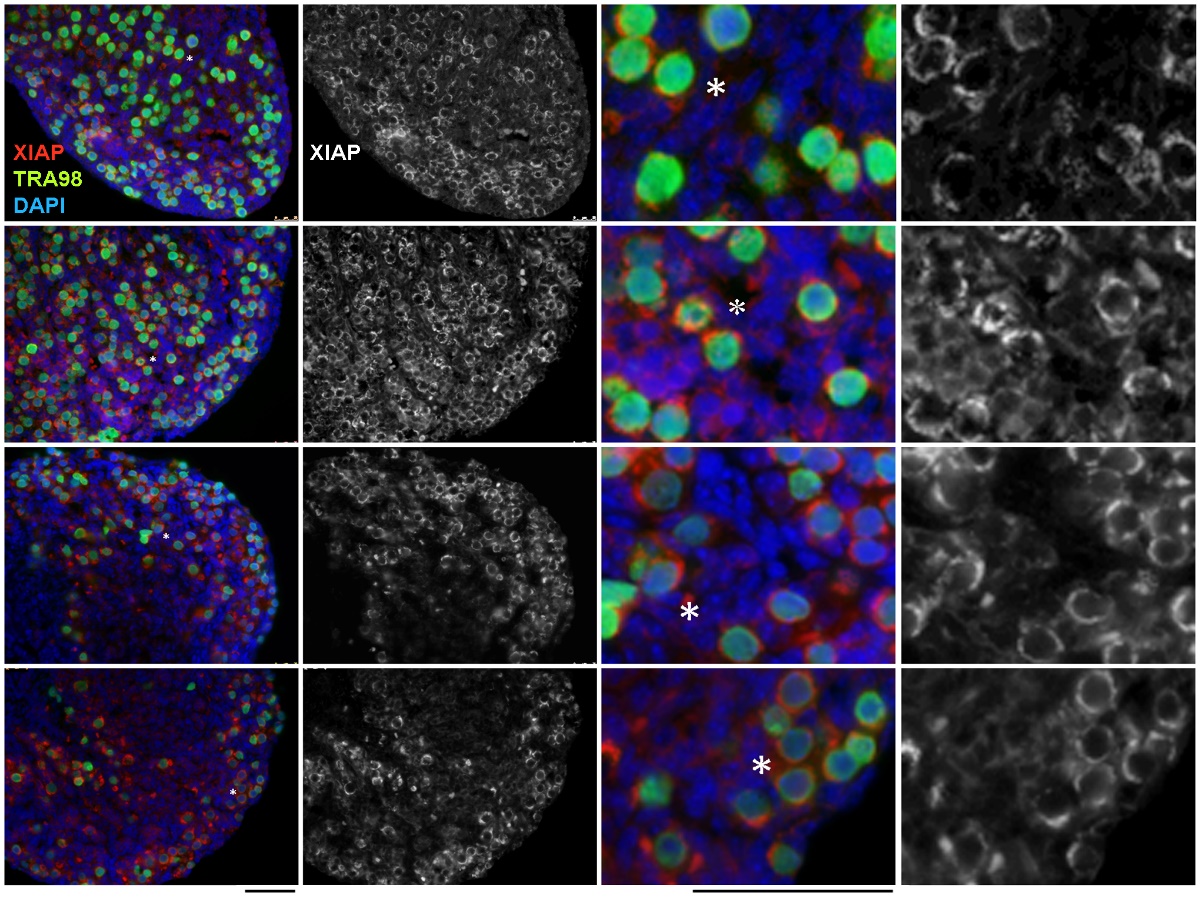


*Xiap-tg*

19.5 dpc

WT

19.5 dpc

*Xiap-tg*

16.5 dpc

WT

16.5 dpc

Fig. S2. XIAP expression in WT and *Xiap-tg* ovaries at 16.5 and 19.5 dpc. **A**. WB showed protein levels of mouse and human XIAP in WT and *Xiap-tg* ovaries at 16.5 dpc detected by anti-XIAP and anti-Myc-tag antibodies at 50 and 75 kDa, respectively. **B**. WB showed protein levels of mouse and human XIAP in WT and *Xiap-tg* ovaries at 19.5 dpc detected by anti-mouse XIAP antibody. **C**. Localization of human and mouse XIAP in WT and *Xiap-tg* ovaries at 16.5 and 19.5 dpc. Ovarian sections were IF-stained for TRA98 (green) and XIAP (red) with DAPI counterstaining (blue). Merged image is followed by XIAP IF-staining alone. Each section is shown at lower (left) and higher (right) magnifications. The asterisk indicates the same position of a section at the two magnifications. Scale bar, 50 μm.

No difference was apparent in ovarian morphology or distribution of Foxl2-positive pregranulosa cells between WT and *Xiap-tg* ovaries at 14.5 to 23.5 dpc.


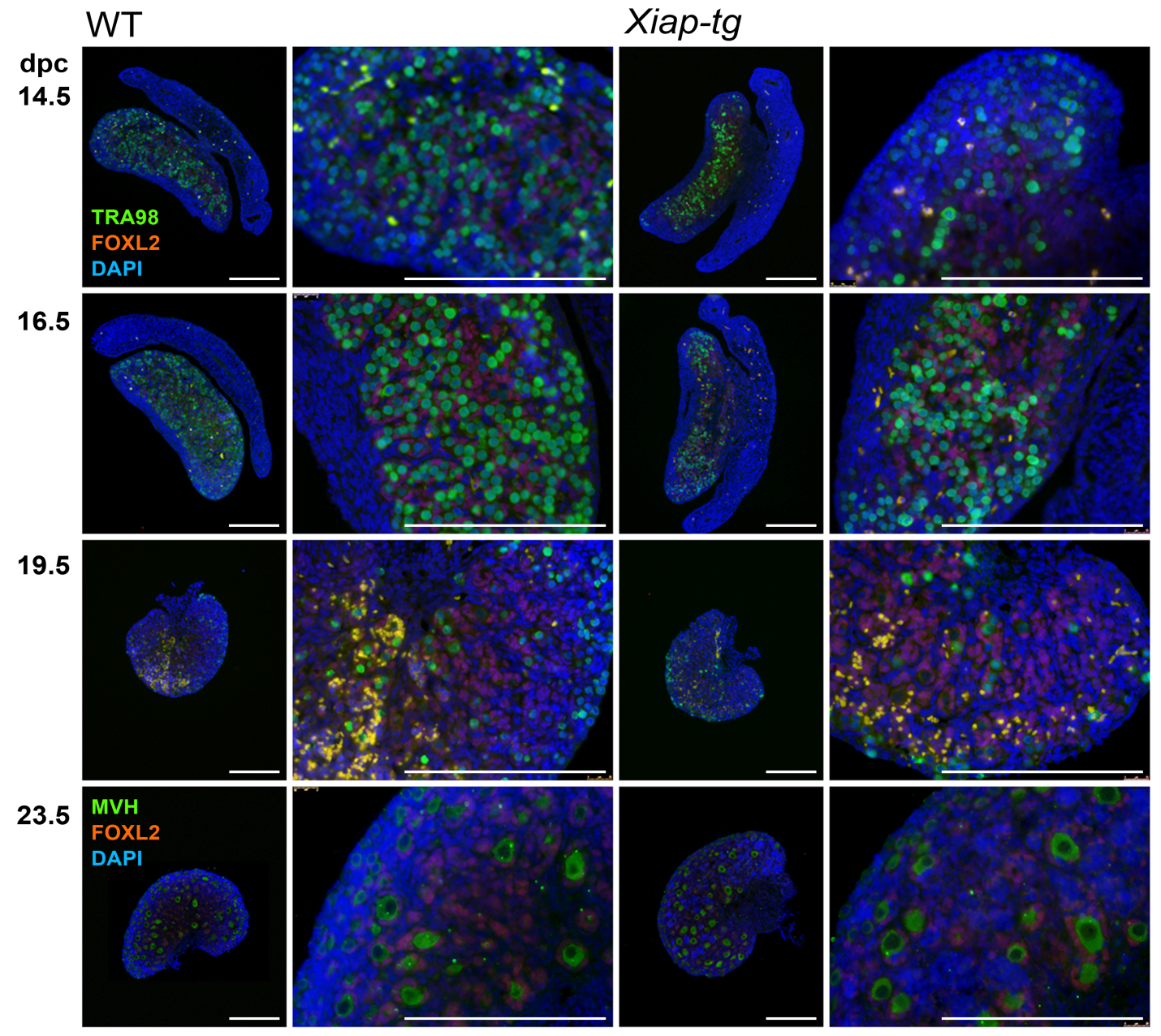


Fig. S3. Ovarian morphology in WT and *Xiap-tg* females at 14.5-23.5 dpc. Ovarian sections were IF-stained for TRA98 or DDX4/MVH (green) and FOXL2 (red) with DAPI counterstaining (blue). Each section is shown at lower (left) and higher (right) magnifications. Scale bar, 400 μm.

No difference was found in transcript levels of *cIap1* or *cIap2* among WT, *Xiap^+/-^*, *Xiap^-/-^*, and *Xiap-tg* ovaries at 18.5 dpc.


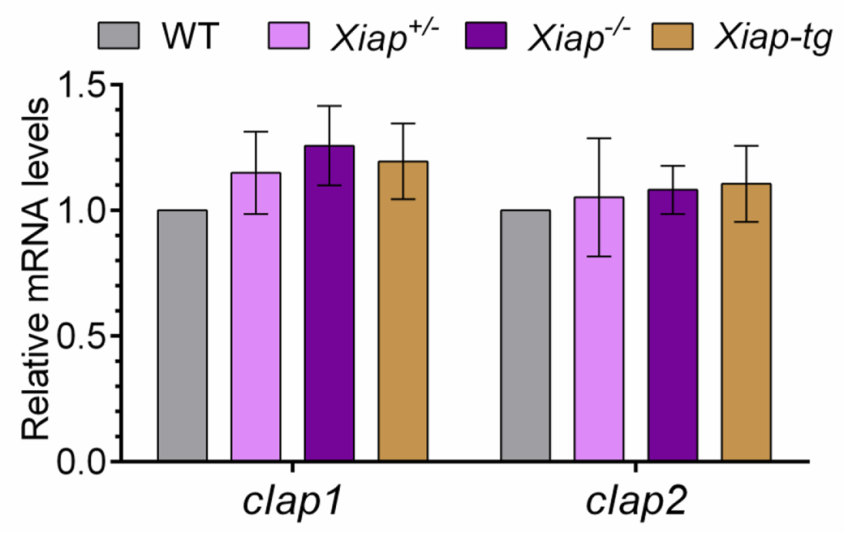


Fig. S4. Transcript levels of *cIap1* and *cIap2* in WT, *Xiap^+/-^*, *Xiap^-/-^* and *Xiap-tg* ovaries at 18.5 dpc. Data are shown as mean ± SEM (n = 3). No significant difference was found among different genotypes by *t*-test.

No difference was found in *Xiap* transcript levels or XIAP proteins levels among *Casp9^+/+^*, *Casp9^+/-^*, and *Casp9^-/-^* ovaries at 16.5 and 18.5 dpc.


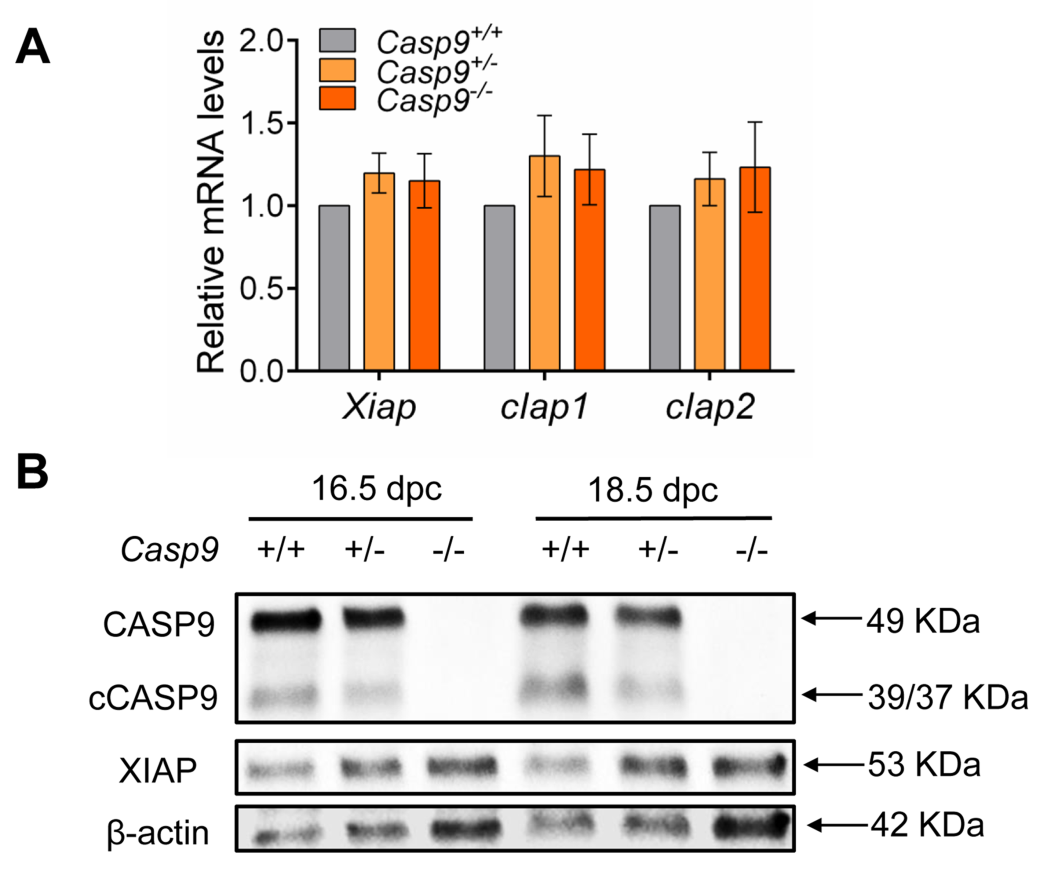


Fig. S5. Expression levels of IAP family genes in *Casp9^+/+^*, *Casp9^+/-^* and *Casp9^-/-^* ovaries at 16.5 and 18.5 dpc. Data are shown as mean ± SEM (n = 3). A. Transcript levels of *Xiap*, *cIap1* and *cIap2* in the ovaries at 16.5 dpc. No significant difference was found among different genotypes by *t*-test. B. XIAP protein levels in the ovaries at 16.5 and 18.5 dpc. CASP9 proteins were undetectable in *Casp9^-/-^* ovaries.

Cleavage levels of CASP9, CASP3, and PARP1 are not affected by a deletion of the XIAP RING-domain, which is required for the degradation of its interacting proteins through ubiquitination.


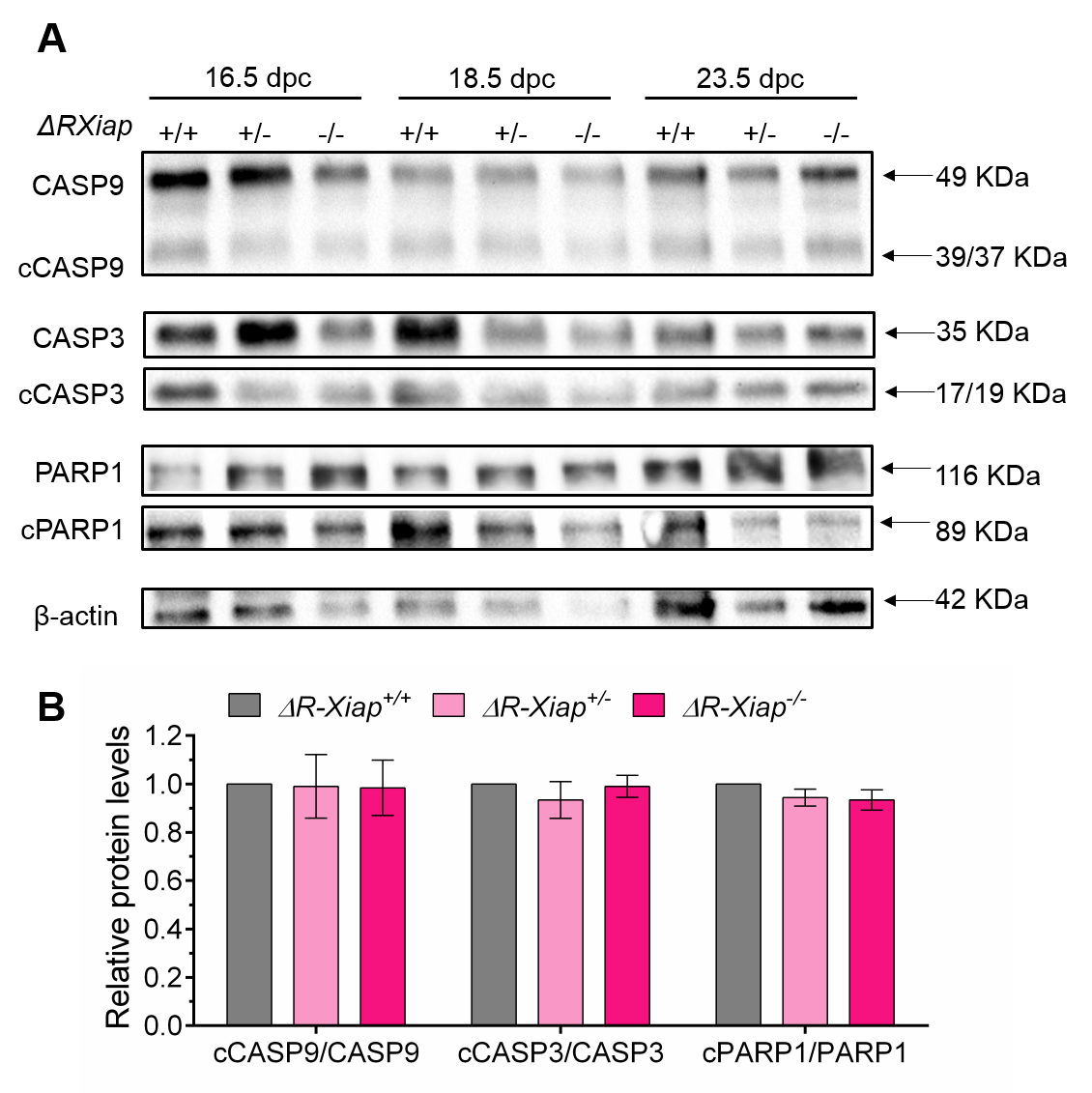


Fig. S6. Cleavage levels of CASP9, CASP3 and PARP1 in *ΔR-Xiap^+/+^*, *ΔR-Xiap^+/-^* and *ΔR-Xiap^-/-^* ovaries at 16.5, 18.5 and 23.5 dpc. **A**. WB of CASP9, CASP3, PARP, and their cleaved forms with β-actin as a loading control. **B**. The ratio of cleaved form to full form of CASP9, CASP3 or PARP1. Data are shown as mean ± SEM (n ≧ 3). No significant difference was found among different genotypes by *t*-test.

RAD51 was enriched along SC axes in the oocytes at the zygotene stage and much fewer at the pachytene stage due to efficient DSB repair in both *Casp9^-/-^* and WT ovaries at 18.5 dpc. RAD51 remained at γH2AFX domains and multiple foci, indicating unsynapsed chromatin regions and incomplete DSB repair, respectively.


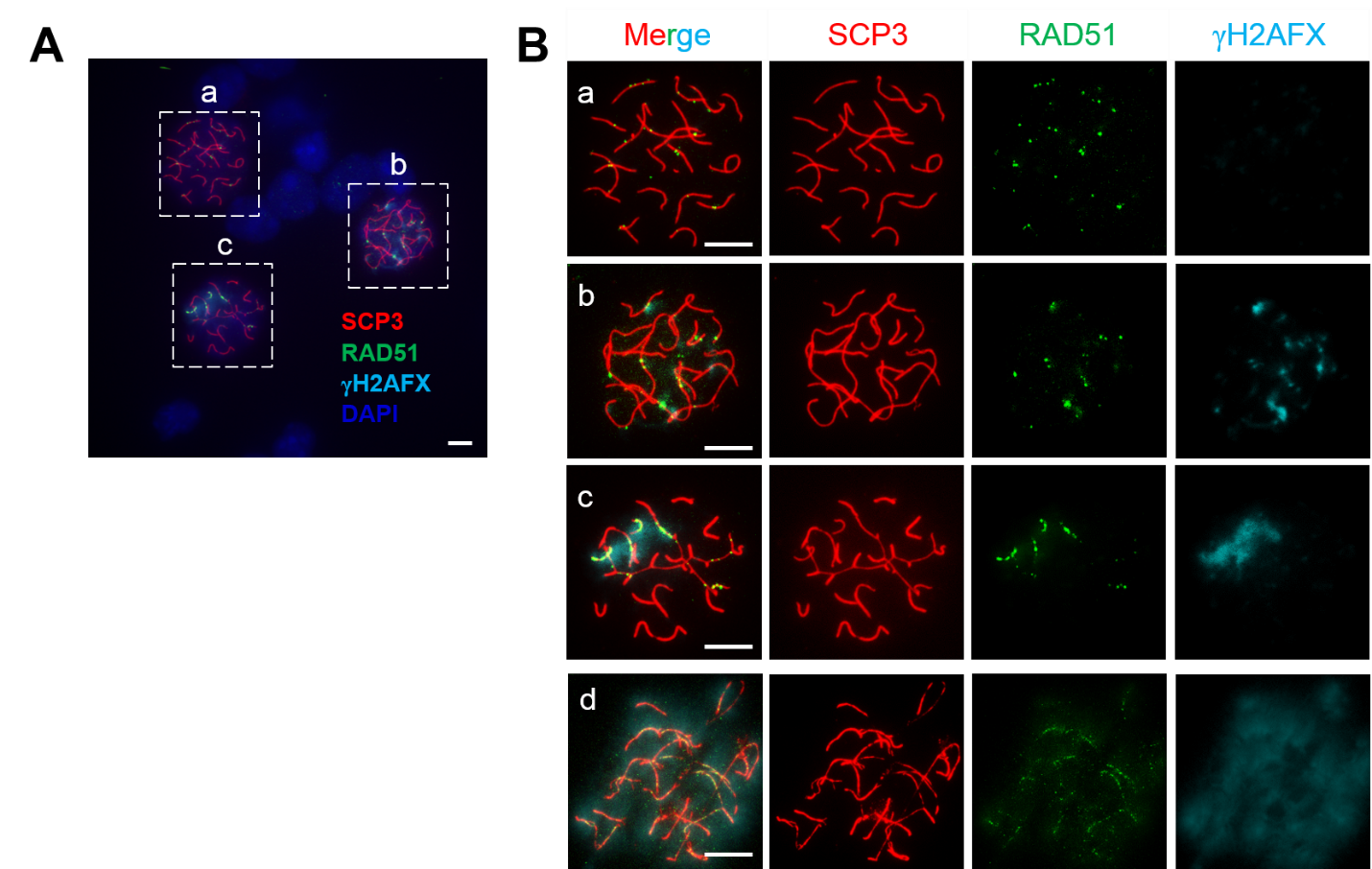


Fig. S7. Localization of RAD51 foci in the oocytes retained in *Casp9^-/-^* ovaries at 18.5 dpc. **A**. Different patterns of γH2AFX accumulation in three pachytene oocytes found in one area of slide. **B**. The oocytes indicated with white dash-line boxes in the left panel are shown at a higher magnification in a-c. **a**. Several RAD51 foci are scattered over SC axes in an oocyte with no γH2AFX signal. **b**. A few RAD51 foci are overlapped with large γH2AFX foci along SC axes. **c**. RAD51 foci are concentrated in the area covered with a large γH2AFX domain. **d** (Not shown in A). Numerous RAD51 foci are seen along SC axes in a zygotene oocyte nucleus covered with a cloud of γH2AFX. Scale bar, 10 μm.
